# Loss of KDM4B impairs osteogenic differentiation of OMSCs and promotes oral bone aging

**DOI:** 10.1038/s41368-022-00175-3

**Published:** 2022-05-07

**Authors:** Peng Deng, Insoon Chang, Jiongke Wang, Amr A. Badreldin, Xiyao Li, Bo Yu, Cun-Yu Wang

**Affiliations:** 1 Laboratory of Molecular Signaling, Division of Oral and Systemic Health Sciences, School of Dentistry, UCLA, Los Angeles, CA USA; 2 Jonsson Comprehensive Cancer Center, UCLA, Los Angeles, CA USA; 3 Section of Endodontics, Division of Regenerative and Reconstructive Sciences, School of Dentistry, UCLA, Los Angeles, CA USA; 4 Division of Preventive and Restorative Sciences, School of Dentistry, UCLA, Los Angeles, CA USA; 5 Department of Bioengineering, Henry Samueli School of Engineering and Applied Science, UCLA, Los Angeles, CA USA

**Keywords:** Oral diseases, Mesenchymal stem cells

## Abstract

Aging of craniofacial skeleton significantly impairs the repair and regeneration of trauma-induced bony defects, and complicates dental treatment outcomes. Age-related alveolar bone loss could be attributed to decreased progenitor pool through senescence, imbalance in bone metabolism and bone-fat ratio. Mesenchymal stem cells isolated from oral bones (OMSCs) have distinct lineage propensities and characteristics compared to MSCs from long bones, and are more suited for craniofacial regeneration. However, the effect of epigenetic modifications regulating OMSC differentiation and senescence in aging has not yet been investigated. In this study, we found that the histone demethylase KDM4B plays an essential role in regulating the osteogenesis of OMSCs and oral bone aging. Loss of KDM4B in OMSCs leads to inhibition of osteogenesis. Moreover, KDM4B loss promoted adipogenesis and OMSC senescence which further impairs bone-fat balance in the mandible. Together, our data suggest that KDM4B may underpin the molecular mechanisms of OMSC fate determination and alveolar bone homeostasis in skeletal aging, and present as a promising therapeutic target for addressing craniofacial skeletal defects associated with age-related deteriorations.

## Introduction

Bone is a dynamic organ that consists of inorganic minerals, organic proteins, cells, lipids, and water and serves essential mechanical and homeostatic functions.^1,2^ However, with aging, the relative proportions of the components change over time. With the deterioration of bony microarchitecture,^2^ a rise in adiposity accompanies the reduction in trabecular bone volume.^3^ As early as age 30, the gradual bone mass reduction may initiate, and dynamic functions of bone become impaired. Bone fragility fracture and delayed healing significantly affect the well being of aging population.^4^ In addition, an imbalance in bone homeostasis and bone-to-fat ratio accumulates with the advance in age, denoting aging as a critical risk factor for developing metabolic disorders such as osteoporosis.^2,3,5^

Aging of the craniofacial skeleton, which is more pronounced in the mandible and alveolar processes,^6^ significantly impedes the repair of trauma-induced bony defects,^7^ and complicates treatment outcomes affecting dental implants and facial esthetics.^8^ The mean cortical porosity, mean cortical width, and bone mass in the mandible showed a parallel correlation between age-related reduction after the age of 50 in both males and females with more significant changes in females.^9^ Also, significant mandibular structure changes occur with aging for both genders, coupled with soft-tissue changes.^10^ Furthermore, various factors such as periodontitis, loss of dentition, diet, diseases, hormone changes, and lifestyle may accelerate aging-related oral bone loss.^2^ Serving as the mechanical anchoring of teeth, alveolar bone loss leads to tooth mobility and tooth loss, significantly diminishing patients’ lifestyle.^2,11^ As the age-related reduction of systemic bone mineral density (BMD) in the axial and appendicular skeleton is related to lower alveolar BMD,^12–14^ skeletal aging in the oral bone could contribute to the age-induced exacerbation of periodontitis. Thus, it is crucial to investigate and understand the molecular factors and their novel mechanisms regulating the oral bone loss to delay or prevent these age-dependent changes.

Mesenchymal stromal or stem cells (MSCs) are multipotent and can give rise to bone, adipose, and cartilage tissue and have therapeutic potential in regenerative medicine.^15,16^ MSCs that are derived from the oral bones (OMSCs) are distinct from long bone marrow-derived MSCs as the mandible develops from neural crest instead of mesoderm.^17^ Substantial studies on the OMSCs revealed when compared to MSCs from the long bone marrow, they possess a distinctively elevated proliferation kinetics, a greater mineralization and osteogenic capacity and a lower adipogenic potential.^18–20^ Since OMSCs can be safely isolated from the human maxillary and mandibular alveolar bone during implant and oral surgeries, they are more suited for craniofacial repair and regeneration.^21,22^ However, little is known about how skeletal aging impacts the renewal and differentiation potentials.

Previously, our lab investigated a novel epigenetic regulatory role of histone lysine demethylase 4B (KDM4B), a histone 3 lysine 9 trimethylation (H3K9me3) demethylase, in skeletal aging and osteoporosis.^3^ Ablation of KDM4B in MSCs promotes cellular senescence and adipogenesis, while inhibiting osteogenesis, suggesting suppression of H3K9me3 may be essential to prevent skeletal aging, MSC exhaustion, and osteoporosis.^3^ However, our previous studies were mainly focused on long bones supporting the vertebrate limb and MSCs isolated from the bone marrow of these bones. Given the differences between the two types of tissues, it requires further exploration to determine whether KDM4B is also engaged in the regulation of oral bone aging. Therefore, we examined the epigenetic mechanism of KDM4B in OMSCs and its regulatory function associated with oral bone homeostasis and oral bone loss induced by aging. We found that loss of KDM4B in OMSCs led to cortical bone thinning and decreased BMD and cancellous bone volume in both aged and ovariectomized (OVX) mice.

## Results

### Loss of KDM4B accelerates age-related oral bone loss

**Fig. 1:**
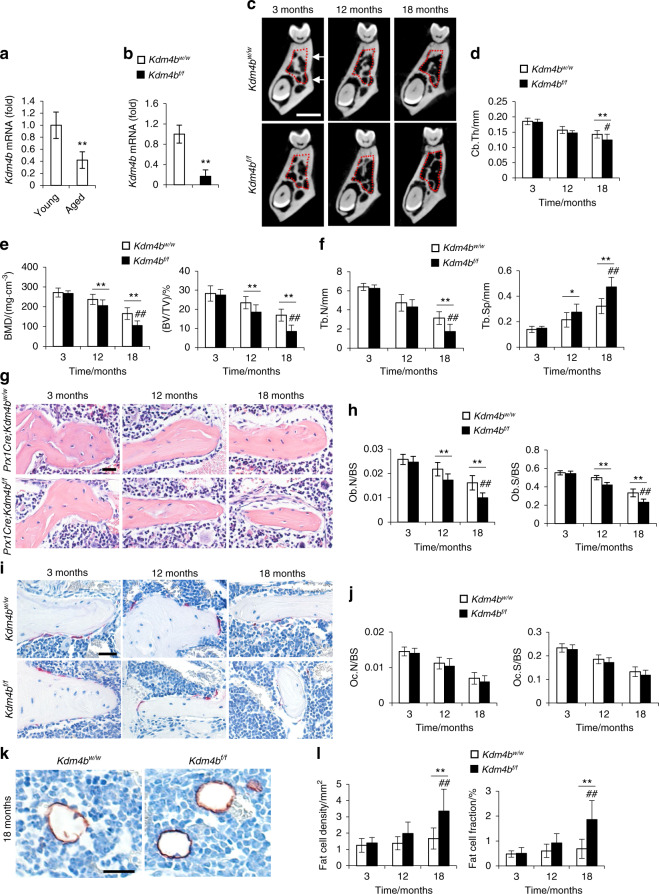
Deletion of Kdm4b in OMSCs enhances age-related bone loss and adipose accumulation in mandible. **a** qRT-PCR showing mRNA levels of *Kdm4b* in OMSCs isolated from young and aged mice. **P < 0.01. *n* = 5. **b** mRNA expression level of *Kdm4b* in OMSCs isolated from *Prx1Cre;Kdm4b*^*w/w*^ (*Kdm4b*^*w/w*^) and *Prx1Cre;Kdm4b*^*f/f*^ (*Kdm4b*^*f/f)*^ mice. **P < 0.01. *n* = 6. **c** Three-dimensional (3-D) images of mandibles constructed by µCT from *Kdm4b*^*w/w*^ and *Kdm4b*^*f/f*^ mice at 3-, 12- and 18-month old, respectively. Scale bar, 1 mm. *n* ≥ 14 per group. Red dashed lines indicate the region of trabecular bone analysis performed, and the arrows indicate the limits for cortical thickness analysis. **d** Quantitative measurements of Cb.Th in mandibles from *Kdm4b*^*w/w*^ and *Kdm4b*^*f/f*^ mice. **P < 0.01; ^#^*P* < 0 .05. *n* ≥ 14 per group. **e** BMD and BV/TV measureme*n*ts by µCT of mandibular bone from *Kdm4b*^*w/w*^ and *Kdm4b*^*f/f*^ mice. **P < 0.01; ^##^*P* < 0.01. *n* ≥ 14 per group. **f** Tb.N and Tb.Sp quantification by µCT of mandibles from *Kdm4b*^*w/w*^ and *Kdm4b*^*f/f*^ mice. *P < 0.05; **P < 0.01; ^##^*P* < 0.01. *n* ≥ 14 per group. **g** Histological hematoxylin and eosin (H&E) staining of trabecular bones in mandibles from *Kdm4b*^*w/w*^ and *Kdm4b*^*f/f*^ mice. Scale bar, 30 µm. *n* ≥ 12 per group. **h** Histological data analysis of osteoblasts number and osteoblasts surface/bone surface (Ob.N/BS and Ob.S/BS, respectively) in mandibles from *Kdm4b*^*w/w*^ and *Kdm4b*^*f/f*^ mice. **P < 0.01; ^##^*P* < 0.01. *n* ≥ 12 per group. **i** TRAP staining of osteoclasts in mandibles from *Kdm4b*^*w/w*^ and *Kdm4b*^*f/f*^ mice. Scale bar, 40 µm. *n* ≥ 12 per group. **j** Quantitative data of Oc.N/BS and Oc.S/BS, respectively, from *Kdm4b*^*w/w*^ and *Kdm4b*^*f/f*^ mice. *n* ≥ 12 per group. **k** Histochemical detection of Perilipin-1 expression in mandibles from *Kdm4b*^*w/w*^ and *Kdm4b*^*f/f*^ mice. Scale bar, 30 µm. *n* ≥ 12 per group. **l** Density of adipocytes and the fat fraction of adipose tissue in mandibles from *Kdm4b*^*w/w*^ and *Kdm4b*^*f/f*^ mice. **P < 0.01; ^##^*P* < 0.01. *n* ≥ 12 per group

To investigate whether KDM4B affects age-related oral bone loss, we obtained OMSCs from the mandibles of both young and aged mice, respectively, and confirmed that *Kdm4b* expression is downregulated in aged OMSCs compared with young OMSCs as determined by quantitative reverse transcriptase polymerase chain reactions (qRT-PCR) (Fig. 1a). For further studies, we performed experiments using *Prx1Cre;Kdm4b*^*f/f*^ (*Kdm4b*^*f/f*^) mice, which specifically delete the expression of KDM4B in MSCs as described in our previous study.^3,23^ Knockout efficiency in OMSCs was confirmed by qRT-PCR (Fig. 1b). To find out whether KDM4B regulates age-related oral bone loss, we harvested the mandibles from 3-, 12-, and 18-month-old *Prx1Cre;Kdm4b*^*w/w*^ (*Kdm4b*^*w/w*^) and *Kdm4b*^*f/f*^ mice and examined with Micro-computed tomography (µCT). 3-D mandibular bone construct at the 1^st^ molar region revealed that 18-month-old *Kdm4b*^*f/f*^ mice presented a significant decrease in the cortical bone thickness (Cb.Th) compared with age-matched *Kdm4b*^*w/w*^ mice; however, there was no significant difference in Cb.Th at 3- and 12-month of age (Fig. 1c, d). BMD of trabeculae and bone volume fraction (BV/TV) of mandibles were also attenuated in *Kdm4b*^*f/f*^ mice than in *Kdm4*^*w/w*^ mice with advanced aging (Fig. 1e). Consistently, we also observed a significant decrease in trabecular bone number (Tb.N) and elevated trabecular spacing (Tb.Sp) in *Kdm4b*^*f/f*^ mice compared to its age-matched control mice at 18-months old (Fig. 1f). Histological analysis revealed the number of osteoblasts (Ob.N/BS) and osteoblast surfaces (Ob.S/BS) relative to total bone surface were decreased in *Kdm4b*^*f/f*^ mice in 12- and 18-month old, compared to their controls (Fig. 1g, h). In contrast, tartrate-resistant acid phosphatase (TRAP) staining demonstrated that osteoclasts’ activity and number were unaffected by the deletion of *Kdm4b* in OMSCs (Fig. 1i, j). Previous studies report that MSCs from long bones could differentiate into both osteoblasts and adipocytes, but the lineage commitment to each cell type is mutually exclusive.^24,25^ Thus, we explored whether loss of bone formation in aged *Kdm4b*^*w/w*^ and *Kdm4b*^*f/f*^ mice led to fat accumulation in the marrow space of the mandible. Although adipogenesis in mandibles is very weak compared with long bones, our immunostaining exhibited a higher fat cell density and fat cell fraction in the mandibles of 18-month-old *Kdm4b*^*f/f*^ mice than 18-month-old *Kdm4b*^*w/w*^ mice (Fig. 1k, l). Taken together, our data infer that loss of KDM4B in OMSCs promotes aging-related oral bone loss.

### KDM4B ablation promotes OVX-induced mandibular bone loss

**Fig. 2:**
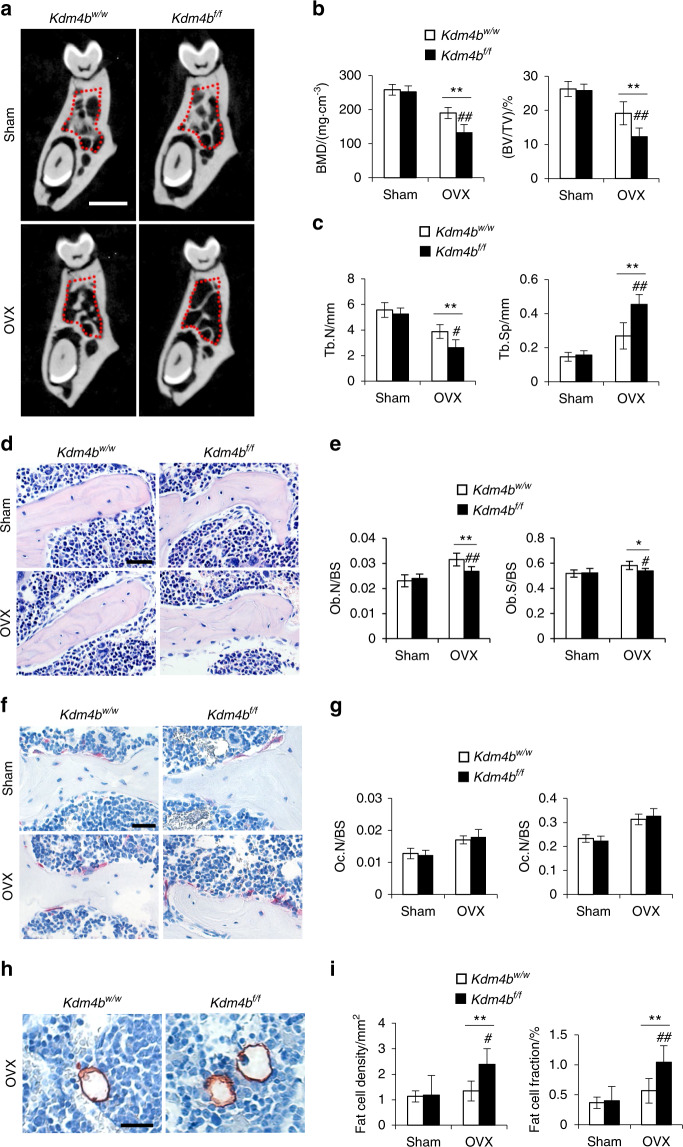
Deletion of Kdm4b in mesenchymal cells using Prx1Cre exacerbates OVX-induced bone loss and adipose accumulation in mouse mandibles. **a** 3-D images of mandibles constructed by µCT from *Kdm4b*^*w/w*^ and *Kdm4b*^*f/f*^ mice after sham and OVX. Red dashed lines indicate the area of trabecular bone analysis performed. Scale bar, 1 mm. *n* = 8. **b** BMD and BV/TV measurements of mandibular bone from *Kdm4b*^*w/w*^ and *Kdm4b*^*f/f*^ mice after sham and OVX. **P < 0.01; ^##^*P* < 0.01^.^ *n* = 8. **c** Tb.N and Tb.Sp quantification of trabeculae of mandibles from *Kdm4b*^*w/w*^ and *Kdm4b*^*f/f*^ mice after sham and OVX. **P < 0.01; ^#^*P* < 0.05; ^##^*P* < 0.01. *n* = 8. **d** H&E staining of trabecular bones in mandibles from sham and OVXed *Kdm4b*^*w/w*^ and *Kdm4b*^*f/f*^ mice. Scale bar, 40 µm. *n* = 8. **e** Quantitative analysis of Ob.N/BS and Ob.S/BS in mandibles from sham and OVXed *Kdm4b*^*w/w*^ and *Kdm4b*^*f/f*^ mice. *P < 0.05, **P < 0.01; ^#^*P* < 0.05; ^##^*P* < 0.01. *n* = 8. **f** TRAP staining of osteoclasts in mandibles from sham and OVXed *Kdm4b*^*w/w*^ and *Kdm4b*^*f/f*^ mice. Scale bar, 40 µm. *n* = 8. **g** Quantitative histological analysis of Oc.N/BS and Oc_._S/BS in mandibles from sham and OVXed *Kdm4b*^*w/w*^ and *Kdm4b*^*f/f*^ mice. **P < 0.01; ^#^*P* < 0.05; ^##^*P* < 0.01. *n* = 8. **h** Histochemical detection of Perilipin-1 expression in mandibles from sham and OVXed *Kdm4b*^*w/w*^ and *Kdm4b*^*f/f*^ mice. Scale bar, 30 µm. *n* = 8. **i** Density of adipocytes and the fat fraction of adipose tissue in mandibles from sham and OVXed *Kdm4b*^*w/w*^ and *Kdm4b*^*f/f*^ mice. **P < 0.01; ^#^*P* < 0.05; ^##^*P* < 0.01. *n* = 8

In addition to bone loss that occurs due to physiological aging, menopause significantly speeds up the bone loss in females. In osteoporotic patients, the decrease in systemic BMD is correlated with a significant reduction in alveolar bone BMD.^13,26^ In this study, we performed OVX in *Kdm4b*^*w/w*^ and *Kdm4b*^*f/f*^ mice and observed the trabecular bone loss in the mandible of both *Kdm4b*^*w/w*^ and *Kdm4b*^*f/f*^ mice; however, the overall BMD, BV/TV, and Tb.N values were lower in OVXed *Kdm4b*^*f/f*^ mice than the OVXed *Kdm4b*^*w/w*^ mice (Fig. 2a–c). In accordance, Tb.Sp were notably higher in OVXed *Kdm4b*^*f/f*^ mice compared to controls and OVXed *Kdm4b*^*w/w*^ mice (Fig. 2c). Deletion of *Kdm4b* significantly reduced the compensatory increase in osteoblasts following OVX, while the increase in osteoclasts was not affected (Fig. 2d–g). Although the induction of adipose tissue accumulation was relatively mild upon OVX, we observed that adipose tissue accumulation was elevated in OVXed *Kdm4b*^*f/f*^ mice with higher adipocyte density and fraction than OVXed *Kdm4b*^*w/w*^ mice (Fig. 2h, i).

### KDM4B intrinsically controls osteogenic differentiation of OMSC

Next, BMSCs and OMSCs were isolated from long bone and mandibles, respectively. Consistent with previous publications, we found OMSCs possessed a stronger osteogenic differentiation potential compared to BMSCs by conducting alkaline phosphatase (ALP) activity and alizarin red S (ARS) staining assay (Fig. S1a–d). Osteogenic gene expressions, including *Col1a1*, *Ibsp*, *Runx2*, *Sp7* and *Bglap*, were also significantly enhanced in OMSCs than BMSCs (Fig. S1e). However, adipogenic differentiation potential of OMSCs was relatively weaker compared with BMSCs (Fig. S1f–h).^18,20,27^ This may explain why we did not observe excessive marrow adipocyte accumulation in mandibles of aged or OVXed mice.

**Fig. 3:**
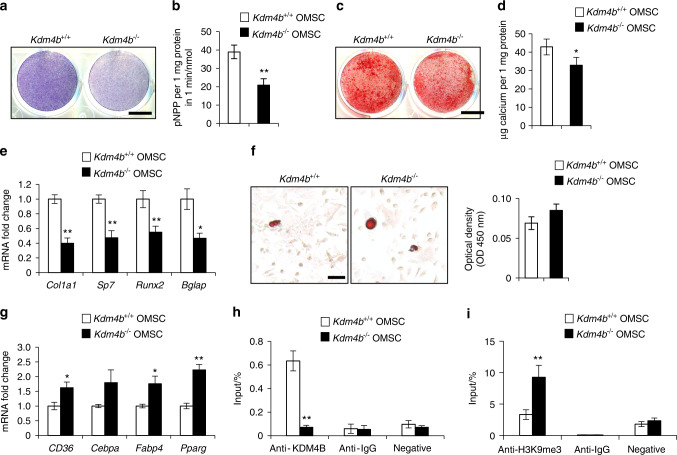
Loss of KDM4B inhibits osteogenic differentiation of OMSCs in vitro. **a** ALP staining of *Kdm4b*^*+/+*^ and *Kdm4b*^*−/−*^ OMSCs undergone osteogenic induction. Scale bar, 8 mm. *n* = 4. **b** Quantitative ALP activity assay of *Kdm4b*^*+/+*^ and *Kdm4b*^*−/−*^ OMSCs upon osteogenic induction. **P *<* *0.01;* *n* = 4. **c** ARS staining of *Kdm4b*^*+/+*^ and *Kdm4b*^*−/−*^ OMSCs undergone osteogenic induction. Scale bar, 8 mm. *n* = 4. **d** Quantification of calcium deposit in ECM of ARS staining of **c**. *P < 0.05; *n* = 3. **e** mRNA expression of osteogenic marker genes *Col1a1, Sp7, Runx2*, and *Bglap* in *Kdm4b* and *Kdm4b*^*−/−*^ OMSCs upon osteogenic induction. *P < 0.05; **P < 0.01. *n* = 3. **f** Oil Red O staining images and quantification of *Kdm4b*^*+/+*^ and *Kdm4b*^*−/−*^ OMSCs following adipogenic induction. Scale bar, 150 μm. *n* = 3. **g** qRT-PCR analysis of the expression of *CD36, Cebpa, Fabp4*, and *Pparg* in *Kdm4b*^*+/+*^ and *Kdm4b*^−/−^ OMSCs upon adipogenic induction. *P < 0.05; **P < 0.01. *n* = 3. **h**, **i** ChIP assays showing the levels of KDM4B (**h**) and H3K9me3 (**i**) on the *Runx2* promoter in *Kdm4b*^*+/+*^ and *Kdm4b*^*−/−*^ OMSCs. **P  < 0.01; *n* = 3

To investigate how KDM4B regulates OMSC differentiation, *Kdm4b*^*+/+*^ and *Kdm4b*^*−/−*^ OMSCs were isolated from *Kdm4b*^*w/w*^ and *Kdm4b*^*f/f*^ mice, respectively. We found that the loss of KDM4B led to the diminished osteogenic differentiation potency in *Kdm4b*^*−/−*^ OMSCs by both ALP activity and ARS staining analysis (Fig. 3a–d). Expression of *Col1a1*, *Sp7*, *Runx2*, and *Bglap* were significantly reduced in *Kdm4b*^*−/−*^ OMSCs than *Kdm4b*^*+/+*^ OMSCs (Fig. 3e). Oil red O staining showed that adipogenic potential of *Kdm4b*^*−/−*^ OMSCs was slightly higher than *Kdm4b*^*+/+*^ OMSCs upon the stimulation with adipogenesis-inducing media which did not reach statistical significance by quantitative measurements (Fig. 3f). Therefore, we further directly examined the adipogenic marker expression with qRT-PCR and determined that *CD36*, *Fabp4*, and *Pparg* levels were elevated in *Kdm4b*^−/−^ OMSCs, compared to *Kdm4b*^*+/+*^ OMSCs (Fig. 3g). Furthermore, as revealed by chromatin immunoprecipitation (ChIP) assays, KDM4B directly bound to *Runx2* promoter, and loss of KDM4B led to an increase in repressive mark H3K9me3 level at *Runx2* promoter in OMSCs (Fig. 3h, i). These results indicate that KDM4B protects the oral bone from an age-induced bone-to-fat imbalance by promoting osteogenic differentiation of OMSCs while inhibiting adipogenesis through *Runx2*.

### Loss of KDM4B in OMSCs attenuates in vivo ectopic bone formation

**Fig. 4:**
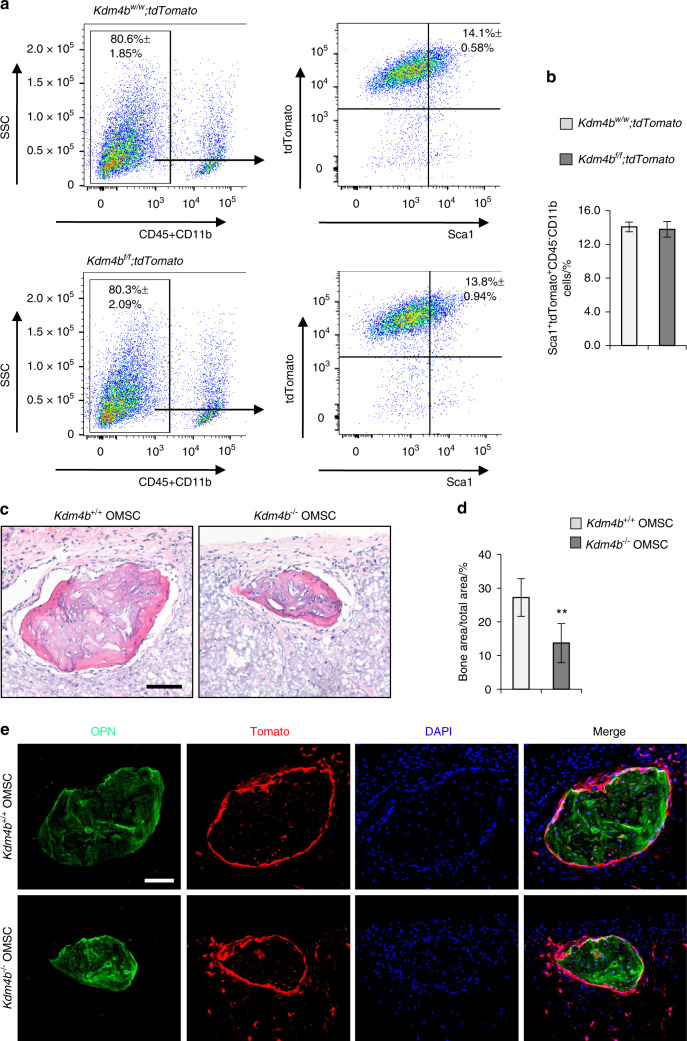
Loss of KDM4B inhibits ectopic bone formation of OMSCs in vivo. **a** FACS analysis for Sca1^+^tdTomato^+^CD45^−^CD11b^−^ OMSCs from *Kdm4b*^*w/w*^*;tdTomato* and *Kdm4b*^*f/f*^*;tdTomato* mice at passage 2. *n* = 3. **b** The percentage of Sca1^+^tdTomato^+^CD45^−^CD11b^−^ OMSCs from (**a**). **c** Histological images of the transplant-induced from *Kdm4b*^*+/+*^ and *Kdm4b*^*−/−*^ OMSCs, respectively. Scale bar, 100 µm. *n* = 9. **d** Quantification of Bone areas over the total areas of transplants. **P < 0.01, *n* = 9. **e** Immunofluorescent images of transplant expressing OPN and tdTomato. Scale bar, 100 µm. *n* = 9

We have generated *Prx1Cre;Kdm4b*^*w/w*^*;tdTomato* (*Kdm4b*^*w/w*^*;tdTomato*) and *Prx1Cre;Kdm4b*^*f/f*^*;tdTomato* (*Kdm4b*^*f/f*^*;tdTomato)* mice allowing us to isolate MSCs through sorting with Sca1^+^tdTomato^+^CD45^−^Cd11b^−^.^3^ We isolated OMSCs (Sca1^+^tdTomato^+^CD45^−^Cd11b^−^) from *Kdm4b*^*w/w*^*;tdTomato* and *Kdm4b*^*f/f*^*;tdTomato* mouse mandibles using flow cytometry (Fig. 4a). The number of OMSCs isolated from *Kdm4b*^*w/w*^*;tdTomato* and *Kdm4b*^*f/f*^*;tdTomato* mice were similar without significant difference at early passages (Fig. 4b). To examine their potential for bone regeneration, we transplanted early-passage OMSCs from *Kdm4b*^*w/w*^*;tdTomato* and *Kdm4b*^*f/f*^*;tdTomato* mice to the dorsal sites of nude mice with a gelfoam scaffold. Mice were sacrificed six weeks after the transplantation and the transplants were collected and examined for histological analysis. Both transplanted OMSCs from *Kdm4b*^*w/w*^*;tdTomato* and *Kdm4b*^*f/f*^*;tdTomato* mice generated bone tissues. However, *Kdm4b*^*−/−*^ OMSCs led to a significantly smaller size of the bone tissues when compared to bone tissues from *Kdm4b*^*+/+*^ OMSCs (Fig. 4c). Also, the quantification of bone areas showed less ectopic bone tissue formation from *Kdm4b*^*−/−*^ OMSCs compared to bone tissues developed from *Kdm4b*^*+/+*^ OMSCs (Fig. 4d). Further, our immunofluorescent staining confirmed that Osteopontin (OPN)-expressing bone tissues were generated, and *Kdm4b*^*+/+*^ OMSCs produced larger bone tissues compared to *Kdm4b*^*−/−*^ OMSCs (Fig. 4e). The expression of tdTomato confirmed that the newly formed bone tissues were generated by the transplanted OMSCs from *Kdm4b*^*w/w*^*;tdTomato* or *Kdm4b*^*f/f*^*;tdTomato* mice. Thus, our findings further demonstrated that KDM4B is an essential histone modifier controlling OMSC-mediated bone formation in vivo.

### KDM4B prevents OMSC senescence

**Fig. 5:**
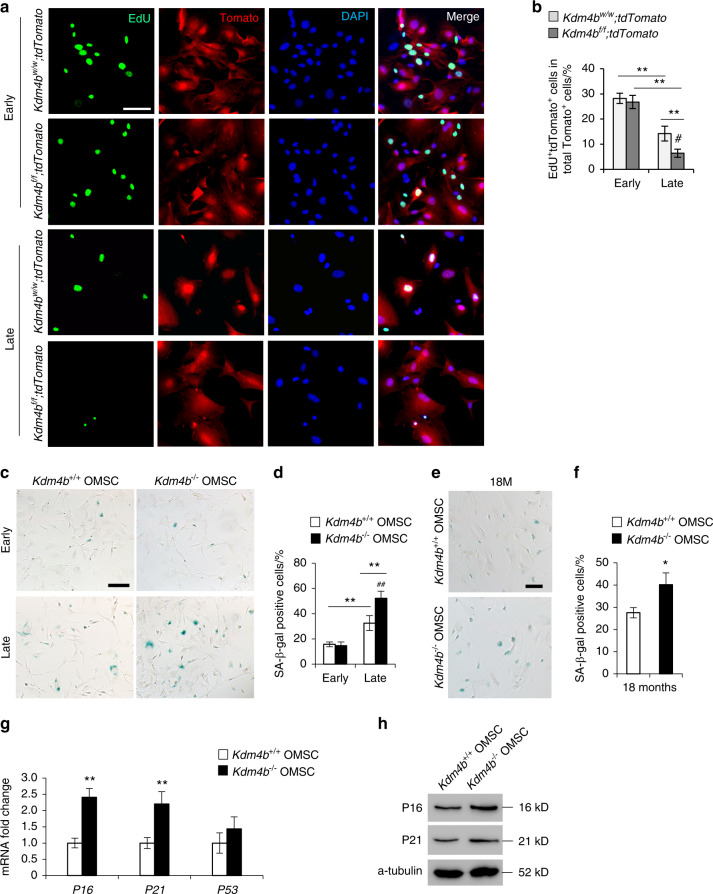
Loss of KDM4B promotes senescence of OMSCs. **a** Microscopic images of EdU incorporation in OMSCs from *Kdm4b*^*w/w*^*;tdTomato* and *Kdm4b*^*f/f*^*;tdTomato* mice at early and late passages, respectively. Scale bar, 40 μm. *n* = 3. **b** Quantification of EdU incorporation assay of (**a**). **P < 0.01; ^#^*P* < 0.05. **c** SA-β-gal staining of *Kdm4b*^*+/+*^ and *Kdm4b*^*−/−*^ OMSCs from 3-month-old *Kdm4b*^*w/w*^ and *Kdm4b*^*f/f*^ mice at early and late passages. Scale bar, 150 μm. *n* = 3. **d** Quantificatio*n* of SA-β-gal staining of (**c**). **P < 0.01; ^##^*P* < 0.01. **e** SA-β-gal staining of *Kdm4b*^*+/+*^ and *Kdm4b*^*−/−*^ OMSCs from 18-month-old *Kdm4b*^*w/w*^ and *Kdm4b*^*f/f*^ mice at passage 1. Scale bar, 150 μm. *n* = 3. **f** Quantification of SA-β-gal staining of (**e**). *P < 0.05. **g** qRT-PCR analyses of the expression of senescence genes markers *P16, P21* and *P53* in *Kdm4b*^*+/+*^ and *Kdm4b*^*−/−*^ OMSCs. **P < 0.01. *n* = 4. **h** Western blot assay showing the expression of P16 and P21 *in Kdm4b*^*+/+*^ and *Kdm4b*^*−/−*^ OMSCs. *n* = 3

Previously, we found that loss of KDM4B leads to increased MSC senescence and skeletal aging in the long bones.^3^ To investigate whether KDM4B also regulates and promotes senescence and aging in OMSCs, we obtained OMSCs from 3-month-old *Kdm4b*^*w/w*^*;tdTomato* and *Kdm4b*^*f/f*^*;tdTomato* mice, and compared their proliferative potential after serial passaging. Interestingly, our results from EDU incorporation assay revealed a reduction in proliferation from early passages (i.e. passages 1-2) to late passages (i.e. passages 8–10), and this reduction is significantly exacerbated in *Kdm4b*^*−/−*^ compared to *Kdm4b*^*+/+*^ OMSCs (Fig. 5a, b). Senescence-associated beta-galactosidase (SA-β-gal) staining showed that *Kdm4b* ablation promoted senescence of late passage OMSCs (Fig. 5c, d). Moreover, *Kdm4b*^*−/−*^ OMSCs from 18-month-old *Kdm4b*^*f/f*^ mice demonstrated significantly increased OMSC senescence at the early passages compared with *Kdm4b*^*+/+*^ OMSCs from the 18-month-old *Kdm4b*^*w/w*^ mice (Fig. 5e, f). In addition, gene expression of *P16* and *P21* were elevated in aged *Kdm4b*^*−/−*^ OMSCs, compared to *Kdm4b*^*+/+*^ OMSCs, while *P53* expression remained unchanged (Fig. 5g). The elevated protein levels of P16 and P21 were validated by western blot analysis (Fig. 5h). Taken together, in addition to influencing the lineage potential, KDM4B also prevents the aging-induced senescence of OMSCs.

## Discussion

Bone is a dynamic mineralized tissue that conducts essential duties in the body, such as support, protection, calcium storage, bone marrow space, and movement.^28^ Bone is refreshed and maintained via a bone remodeling process that conserves the sophisticated homeostatic balance between bone resorption and formation.^29^ Bone-marrow MSCs are a critical progenitor source that give rise to osteoblasts, osteocytes, and chondrocytes.^30,31^ Orchestration of the osteogenic potential in MSCs is tightly regulated to optimize bone development, repair, and regeneration. However, aging induces senescence and significantly alters the lineage propensities of MSCs, resulting in diminished stemness and aberrant lineage commitment. As a result, aged bones exhibit significant reduction in overall bone volume and bone mass regardless of gender or ethnicity, entailing considerable risks for fracture, osteoporosis, and diminished functions.^32^ Interestingly, our lab has revealed that the histone demethylase KDM4B promotes the osteogenic commitment of MSCs while inhibiting adipogenic differentiation, indicating that epigenetic regulation may play a vital role in determining the MSC cell fate in skeletal aging and metabolic bone diseases.^33^

Growing evidence suggests that aging affects the skeletal system by modulating the behavior of MSCs and promoting their exhaustions.^2,34^ Bork et al. observed DNA methylation pattern changes in MSCs that have undergone replicative senescence and aging and suggested that specific epigenetic modifications may regulate MSC aging.^35^ Many other studies also affirmed that epigenetic regulation affects MSC aging and cell fate determination.^36,37^ It is well established that senescent MSCs undergo phenotype changes that result in diminished stemness and osteogenic differentiation potential, affecting the bone mass and repair capacity of long bones.^38–40^ In this study, we showed that the loss of KDM4B in OMSCs inhibits osteogenesis and promotes OMSC senescence in the aged murine model. Mechanistically, we revealed the epigenetic effect of histone demethylase KDM4B on *Runx2* promoter in modulating downstream gene expression. RUNX2 is a well-known transcription factor of osteogenesis and has been shown to repress adipogenesis.^41^ Our data illustrated that KDM4B regulates H3K9me3 expression at the promoter region of *Runx2* to modulate the gene expression, resulting in decreased osteogenesis and promoting adipogenic differentiation in OMSCs, contributing to the decreased bone formation in aged mandible. Our findings here demonstrate a novel epigenetic regulation of MSC cell fate determination and senescence in the craniofacial skeleton.

Interestingly, age-dependent changes in bone morphology, mass, BMD, and volume also occur in oral jawbones, primarily in the mandibular alveolar bone.^3,42^ These bony changes are exacerbated in the presence of periodontitis and loss of dentition, increasing the risk of fractures and diminishing essential daily oral functions, including mastication and the ability of maintaining a bony framework for esthetics.^3,43^ Strong clinical evidence supports significant correlations between alveolar bone density and other skeletal sites such as hip or vertebral BMD.^44,45^ Nonetheless, a direct causal association between osteoporosis and mandibular bone loss has not yet been established. Here we show that the loss of KDM4B in OMSCs significantly exacerbated the oral bone loss in both physiological aging and OVX mouse models. Our findings suggest that maintaining KDM4B level in OMSCs may prevent or delay mandibular bone aging in old and/or osteoporotic patients. However, further investigations are necessary to identify and illustrate the potential function of KDM4B in alveolar bone repair, especially in aged bone, that may reinforce the importance of the KDM4B as a therapeutic target for regenerative medicine and to prevent skeletal aging.

Similar to our previous findings in long bone-marrow-derived MSCs,^3^ loss of KDM4B in OMSCs led to reduced osteogenesis, resulting in an exacerbated bone loss in the mandible of the aged mice. However, unlike the femoral bone marrow, a significant difference between marrow adipose tissue was only detected in the mandibles of 18-month-old *Kdm4b*^*w/w*^ and *Kdm4b*^*f/f*^ mice. Adipogenesis of *Kdm4b*^*+/+*^ and *Kdm4b*^*−/−*^ OMSCs in vitro seem diminished compared to *Kdm4b*^*+/+*^ and *Kdm4b*^*−/−*^ MSCs harvested from the femoral bone marrow. The reason for this inconsistency may be in the case that MSCs derived from different tissues possess distinct osteogenic, chondrogenic, and adipogenic differentiation potentials. Yamaza et al. also demonstrated that mouse MSCs harvested enzymatically from the mandible presented a higher osteoblastic differentiation potency than bone marrow-derived MSCs from the long bone and indicated that OMSC is a distinct population of cells that has unique differentiation characteristics and immunomodulation abilities.^46^ Moreover, Aghaloo et al. also demonstrated that OMSCs have increased osteogenic potential, and augmented OMSCs implanted into nude mice produced 70% larger bone nodules with three-fold increase in mineralized bone formation than long-bone derived-MSCs, suggesting that OMSCs exhibit distinct mechanisms to regulate mandible homeostasis and may contribute to the pathophysiology of disease unique to the jaw.^18^ In this study, although loss of KDM4B affected adipogenic differentiation, we did not observe a drastically enhanced adipose tissue accumulation in the oral bones compared with the long bones which also may be due to the intrinsic differences between MSCs derived from oral and long bones. In conclusion, our study presents important understanding of the protective effect of epigenetic regulation on oral bone aging through the histone demethylase KDM4B.

## Materials and methods

### Mice

*Prx1Cre;Kdm4b*^*w/w*^ and *Prx1Cre;Kdm4b*^*f/f*^ mice were generated as previously described.^3^ All mice were housed in the Division of Laboratory Animal Medicine (DLAM) at UCLA following the DLAM protocols and US National Institute of Health guidelines. For our mandibular aging studies, both male and female mice were randomly assigned to each procedure group at a sample size of at least 12 mice per group, respectively. For OVX, 8 female mice per group were used. The primer sequences for genotyping are as follows: *Kdm4b*, 5′-ACCCAGGACTGATGTTCACA-3′ and 5′-GAAAGAAGACCTGAGCTGTC-3′; *Cre*, 5′-GCATTACCGGTCGATGCAACGAGTGATGAG-3′ and 5′-GAGTGAACGAACCTGGTCGAAATCAGTGCG-3′; *tdTomato* (wildtype), 5′-AAGGGAGCTGCAGTGGAGTA-3′ and 5′-CCGAAAATCTGTGGGAAGTC-3′; and *tdTomato* (tdTomato), 5′-GGCATTAAAGCAGCGTATCC-3′ and 5′-CTGTTCCTGTACGGCATGG-3′. In vivo transplantation of OMSCs from *Prx1Cre;Kdm4b*^*w/w*^*;tdTomato* and *Prx1Cre;Kdm4b*^*f/f*^*;tdTomato* mice were performed as previously described.^3^ 100 µL of cell suspension with approximately 1 × 10^6^ cells were incubated with Gelatin gelfoam scaffold (Ferrosan) at 37 °C for 6 h. The scaffolds with OMSCs were then transplanted subcutaneously at the dorsal sites of nude mice (*n* = 9). 6 weeks after transplantation, mice were sacrificed, and the scaffolds were collected and fixed with 4% paraformaldehyde for histological analysis. The area of mineralized tissue compared to the total area were measured using SPOT 4.0 software (Diagnostic Instruments).

### Cell culture and flow cytometry

OMSCs were isolated from 3-months old (young) and 18- to 24-month old (aged) mice. Mice were euthanized with CO_2_ until breathing stopped, and cervical dislocation confirmed the death. Mice were then sterilized with 70% ethanol. Skins, skeletal muscle, and connective tissues were all dissected and removed, and the bilateral mandibular bones were collected for further experiments. Mandibles were subjected to enzymatic digestion via 3 mg·mL^−1^ collagenase type I (Worthington Biochem) and 4 mg·mL^−1^ dispase II (Roche Diagnostic) for 60 min at 37 °C to collect OMSCs. BMSCs were isolated from the bone marrow by flushing the long bones using syringes with a 26-gauge needle. Collected bone marrows were incubated with 3 mg·mL^−1^ of Collagenase Type I (Worthington) and 4 mg·mL^−1^ Dispase (Roche diagnostics) for 30 min at 37 °C. After washing with PBS 2 times, cells were seeded into cell culture dishes and were allowed to attach for 2–3 days at 37 °C in a humidified 5% CO_2_ incubator maintained. The medium was comprised of Dulbecco’s Modified Eagle Medium (DMEM), supplemented with 15% FBS and 1% penicillin/streptomycin (all from Gibco). After 12–14 days of culture, the cells were passaged using trypsin (Sigma-Aldrich). For flow cytometry analysis, single-suspension cells were collected using 70-µm cell strainers (BD Biosciences) and stained with fluorochrome-conjugated antibodies for 30 min on ice in the dark. Antibodies were diluted with PBS with 2% FBS. FITC-conjugated anti-mouse Sca1 (1:100 dilution), APC-conjugated anti-mouse CD45 (1:100 dilution), and APC-conjugated anti-mouse CD11b (1:100 dilution) were all purchased from Biolegend, following the manufactures recommended protocol to perform experiments. Subsequently, sorted cells and data were collected with a BD FACS Aria III and integral Flow software (version 10.5.3) by the UCLA Flow Cytometry Core. Isolated OMSCs and BMSCs were cultured in a humidified 5% CO_2_ incubator at 37 °C in Dulbecco’s Modified Eagle Medium (Gibco) with 15% FBS (Gibco) and 1% penicillin/streptomycin (Gibco).

### ALP, ARS, and Oil-Red O Staining

For osteoblastic induction, the inducing media was prepared with 100 µmol·L^−1^ ascorbic acid (Sigma-Aldrich), 2 mmol·L^−1^ β-glycerophosphate (Sigma), and 10 nmol·L^−1^ dexamethasone (Sigma-Aldrich) in α-MEM (Gibco) with 10% FBS and 1% penicillin/streptomycin. For adipogenic induction, the inducing media were prepared by adding 1 µmol·L^−1^ dexamethasone (Sigma-Aldrich), 10 µg·mL^−1^ insulin (Sigma-Aldrich), 0.5 mmol·L^−1^ 3-isobutyl-1-methylxanthine (Sigma-Aldrich), and 0.2 mmol·L^−1^ indomethacin (Sigma) in α-MEM (Gibco) with 10% FBS and 1% penicillin/streptomycin. OMSCs within 10 passages were used for cell senescence studies considering passages 1 to 2 as early passages and passages 8–10 as late passages.

For ALP staining, cells were cultured in 12-well plates with the osteogenic induction medium for 5–7 days (media was refreshed every 2–3 days during this period of time) once more than 90% confluence are achieved. First, we fixed cells with 70% ethanol for 15 min at room temperature (RT) after removing the media, followed by staining for 30 min at 37 °C with a fresh-made ALP staining buffer composed of 0.25% naphthol AS-TR phosphate (Sigma-Aldrich) and 0.75% Fast Blue BB salt (Sigma-Aldrich) in 0.1 mol·L^−1^ Tris buffer solution (pH 9.6). ALP activity was quantified by measuring and recording the absorbance of the mixture consisting 20 µL of cell extract, 50 µL ALP stabilizing buffer (Sigma-Aldrich), 50 µL pNPP liquid substrate (Sigma-Aldrich) and incubated for 20 mins at 37 °C at OD405 nm using CLARIOstart Plus (BMG Labtech). For ARS staining, cells were cultured for 2–3 weeks with osteoblastic induction media, then fixed with 70% ethanol. Consequently, fixed cells were incubated with 1% ARS solution (Sigma-Aldrich) for 5 min at RT, and the staining was scanned and saved with HP Scanjet G4050 (HP). For ARS quantification, plate was destained with 10% cetrlpyridinium chloride solution (Sigma-Aldrich) and measured the absorbance at 562 nm for recording and analysis. For Oil-Red O Staining, cells were cultured in 12-well plates and adipogenic induction medium was added for 2–3 weeks (media was refreshed every 2–3 days) after more than 90% confluency was confirmed. Cells were washed with 1X PBS, and then fixed with 70% Ethanol for 10 min. Oil Red O staining solution was prepared as follows: 3 parts of Oil Red O stock solution was added to 2 parts of ddH_2_O, mixed well, and then filtered with a 0.45 µmol·L^−1^ filter. After fixative solution was removed, cells were washed with 60% isopropanol, and then incubated with Oil Red O working solution to each well for 10 min. After confirming lipid droplets staining under the microscope, staining solution was removed and cells were washed twice with the tap water. Images were taken under the microscope within 24 h of staining then subjected for quantification. Absorbance was measured and recorded at OD450 nm using a microplate reader (Bio-Rad).

### EdU incorporation assay and SA-β-gal staining

Cells were cultured on 8 chamber cell culture slides (Corning) and incubated with 10 µmol·L^-1^ EdU for overnight at 37 °C. After incubation, cells were fixed with 4% paraformaldehyde for 15 min at RT, then washed with 3% BSA in PBS. Subsequently, the cells were incubated with 0.5% Triton X-100 in PBS at RT for 20 min. Click-IT reaction cocktails (Invitrogen) were added to the cells for 15 min at RT in the dark and counterstained with ProLong Diamond Antifade Mountant with DAPI (Invitrogen). Coverslips were placed, and EdU levels were measured using click-it EdU alexa fluor 488 imaging kit (Thermo Fisher Scientific). Both EdU-positive and negative cells were counted in at least 20 different image fields, and the percentage of EdU-positive cells was calculated.

For SA-β-gal staining, OMSCs were cultured in 6-well plates and stained with SA-β-gal cell staining kit (Cell Signaling Technology). Cells were fixed with fixative solution for 10 min at RT. β-gal staining solution was added and incubated at 37 °C overnight. Cells with SA-β-gal activity were counted in at least 20 different image fields. SA-β-gal activity was analyzed in *Kdm4b*^*+/+*^ *and Kdm4b*^*−/−*^ OMSCs in early (passages 1–2) and late passages (passages 8–10), respectively.

### Immunohistochemistry and immunofluorescence staining

The specimens were decalcified with 10% ethylenediaminetetraacetic acid (EDTA) at pH 7.4 for 2 weeks at RT and sent to UCLA Translational Pathology Core Laboratory (TPCL) for paraffin embedding and sectioning (5 µm) for staining. Sections were incubated at 65 °C for 2 h and deparaffinized with 100% xylene twice for 5 min followed by the series of rehydration steps with distilled water and ethanol (100% to 20%). Subsequently, for antigen retrieval, slides were incubated with 10 mmol·L^−1^ Sodium Citrate acid buffer at pH 6.0 and placed in the pressure cooker until 20 psi was reached, then cooled to the RT. Slides were stained with peroxidase block (Dako EnVision System) for 10 min, washed with PBS for 5 minutes three times, and incubated with the 1:100 concentration of rabbit polyclonal anti-mouse Perilipin-1 antibody (Cell signaling Technology) diluted with Antibody Diluent (Dako EnVision System) overnight at 4 °C. Slides were washed and stained with Envision+HRP-labeled polymer (Dako EnVision System) at RT. Immunocomplex expression was determined using AEC + chromogen (Dako EnVision System), and hematoxylin QS (Fisher) was used to counterstain the slides.

For immunofluorescence staining, bone tissues were fixed in 4% paraformaldehyde and placed in 10% EDTA pH 7.4 for 2 weeks at RT for decalcification. After decalcification, bone tissues were incubated in 30% sucrose for a minimum of 48 h at 4 °C in the dark and prepared for the frozen section at UCLA TPCL (5 µm per slide). Frozen section slides were blocked with PBS solution containing 10% horse serum for 30 min, and then stained with 1:100 concentration of primary antibody (Mouse OPN antibody, R&D System) overnight at 4 °C. Slides were then incubated with the 1:200 secondary antibodies in the dark for 1 h at RT. The secondary antibody for OPN (AffiniPure Donkey Anti-Goat IgG) was purchased from Jackson ImmunoResearch Inc. Slides were then counterstained with 4′6-diamidino-2-phenylindole (DAPI; Thermo Fisher Scientific) and covered with an anti-fade mounting medium (Thermo Fisher Scientific). Images were taken at random 20 places with an Olympus IX 51 microscope for analysis.

### qRT-PCR

Cells were cultured and washed with PBS before collection. Total RNA was isolated using TRIzol reagent (Invitrogen) and NucleoSpin RNA kit (Macherey-Nagel). To synthesize cDNA, 1 µg of RNA from each sample was mixed with 1 µL of random hexamer (50 µmol·L^−1^, Invitrogen) and 1 µL of dNTP (10 mmol·L^−1^, Promega) at 70 °C for 5 min and placed on ice. In the same tube, a mixture of 2 µL of 10X M-MuLV buffer (NEB), 0.2 µL of RNase Inhibitor (40 U·µL^−1^, NEB), and 1 µL of MuLV reverse Transcriptase (200 U·µL^−1^, NEB) was added and incubated at 42 °C for 1 h and 85 °C for 5 min for cDNA synthesis. cDNA reaction diluted 1:40 was mixed with 0.2 µmol·L^−1^ forward and reverse primers and iQ SYBR Green Supermix (Bio-Rad) containing dNTPs, iTaq™ DNA Polymerase, MgCl_2_, SYBR^®^ Green I, enhancers, stabilizers, and fluorescein. qRT-PCR was performed on an CFX96 real time PCR detection system (Bio-Rad) with reactions at 95 °C for 1 min followed by 40 cycles of 95 °C for 10 s, 60 °C for 1 min. qRT-PCR data was analyzed using CFX Manager Software Ver. 3.1 (Bio-Rad). The primers for qRT-PCR are as follows: *Gapdh*, 5'-ACAACTTTGGCATTGTGGAA-3' and 5'-GATGCAGGGATGATGTTCTG-3'; *Col1a1*, 5′-TAGGCCATTGTGTATGCAGC-3′ and 5′-ACATGTTCAGCTTTGTGGACC-3′; *Ibsp*, 5′-ATGGAGACGGCGATAGTTCC-3′ and 5′- CTAGCTGTTACACCCGAGAGT-3′; *Sp7*, 5′-ATGGCGTCCTCTCTGCTTG-3′ and 5′-TGAAAGGTCAGCGTATGGCTT-3′; *Runx2*, 5′-TCCACSSGGACAGAGTCAGATTACAG-3′ and 5′-CAGAAGTCAGAGGTGGCAGTGTCATC-3′; *Bglap*, 5′-CTGACCTCACAGATGCCAAGC-3′ and 5′-TGGTCTGATAGCTCGTCACAAG-3′; *CD36*, 5′-GGACATTGAGATTCTTTTCCTCTG-3′ and 5′-GCAAAGGCATTGGCTGGAAGAAC-3′; *Cebpa*, 5′-GCAAAGCCAAGAAGTCGGTGGA-3′ and 5′-CCTTCTGTTGCGTCTCCACGTT-3′; *Fabp4*, 5′-AAGGTGAAGAGCATCATAACCCT-3′ and 5′-TCACGCCTTTCATSSCACATTCC-3′; *Pparg*, 5′-GTACTGTCGGTTTCAGAAGTGCC-3′ and 5′-ATCTCCGCCAACAGCTTCTCCT-3′; *P16*, 5′-TGTTGAGGCTAGAGAGGATCTTG-3′ and 5′- CGAATCTGCACCGTAGTTGAGC-3′; *P21*, 5′-TCGCTGTCTTGCACTCTGGTGT-3′ and 5′- CCAATCTGCGCTTGGAGTGATAG-3′; and *P53*, 5′-ATGGCCATCTACAAGAAGTCACAG-3′ and 5′- ATCGGAGCAGCGCTCATG-3′.

### Western blot analysis

Cells were collected, lysed and extracted using whole cell lysis buffer (Sigma-Aldrich) with 1:100 volume of protease inhibitor cocktail for 1 h on ice. The protein concentration was measured colorimetrically using Bio-Rad reagents. 40 µg of total proteins were resolved, separated on 12.5% SDS-PAGE, and then transferred onto a polyvinylidene difluoride (PVDF) membrane using a Bio-Rad semidry transfer system. After the transfer, membrane was blocked with 5% dry-milk in TBS/Tween20 (TBST) buffer at RT for 1 h and incubated with primary antibodies diluted in TBST buffer with 5% dry-milk at 4 °C overnight. After washing, secondary antibodies diluted in TBST with 5% dry-milk were introduced for 2 h at RT (Anti-rabbit-HRP: 1:7 500, Promega and Anti-mouse-HRP: 1:5 000, Promega) and detected using ECL reagents (Pierce) and Bio-Rad ChemiDoc MP system. Primary antibodies used in this study are and α-tubulin (1:10 000; Sigma-Aldrich), P16 (1:2 000; Cell Signaling Technology) and P21 (1:2 000; Cell Signaling Technology).

### Chromatin immunoprecipitation (ChIP) assay

Cells were collected using cell scrapers and then incubated with 5 nmol of dimethyl 3,3′ dithiobispropionimidate-HCl (DTBP, Fisher) for 10 min at RT followed by the treatment with 1% formaldehyde for 15 min in a 37 °C water-bath. Minimum of 2 × 10^6^ cells were used for each ChIP reaction. ChIP assays was performed using a ChIP assay kit (Upstate), and the amount of the precipitated DNAs were evaluated with qPCR and calculated as the percentage of input DNA.^47,48^ Antibodies for ChIP assays were purchased from the following commercial sources: anti-KDM4B (Bethyl laboratories; 1:200 dilution), anti-H3K9me3 (Abcam; 1:500 dilution), and anti-IgG (Cell Signaling Technology; 1:500 dilution). The primary sequence used in ChIP assays are: *Runx2* promoter region: Forward (5′-TGCCACCGACTGAGAGAA-3′) and Reverse (5′-CAGGCTACTGAATTCATTTCTTG-3′) and negative region: forward (5′-GACTCCTTTCAGGCACCATC-3′) and Reverse (5′-GCTGTTAGGCTCGAGGATTG-3′).

### µCT analysis

The mandibles of 3-, 12-, and 18-month old *Prx1Cre;Kdm4b*^*w/w*^ and *Prx1Cre;Kdm4b*^*f/f*^ mice were obtained and dissected. The specimens were oriented, and µCT analysis was performed using a Skyscan 1275 µCT imaging system (Skyscan, Kontich, Belgium) with a spatial resolution of 11 µm at 60 kV/166 µA; 360 rotation model; 1 mm filter applied. NRecon 1.6 software was used to evaluate volumetric reconstructions, and the analysis and calculation were performed with CTAn 1.15 software. Bone volume (BV/TV, %), BMD, Tb.N, and Tb.Sp were calculated within the delimited volume of interest. For trabecular bone analysis, the volume of interest was defined for the area enclosed between the mesial and distal root of the first mandibular molar below the furcation zone on a buccal-lingual cross-section of the molar. For cortical bone thickness analysis, the volume of interest defined the lingual side of the cortical bone extending from the furcation area to coronal of the embedded incisor on a buccal-lingual cross-section of the first mandibular molar.

### Quantification and statistical analysis

Statistical analysis used in this study is the two-tailed unpaired Student’s *t* test and two-way ANOVA with Holm–Sidak posthoc test. For the calculation, SPSS 23.0 software was used. *P*-values < 0.05 were considered as statistically significant unless specifically indicated (*P < 0.05 and **P < 0.01 for between groups; ^#^*P* < 0.05 and ^##^*P* < 0.01 for age or OVX x genotype interaction). All data were analyzed as the mean ± standard deviation (mean ± SD), and the data were collected from at least three independent experiments/samples.

## Supplementary information

Supplemental Material

## Data Availability

The authors are exclusively responsible for the content of this article and datasets collected to support the findings of this study. The data for this study are available from the corresponding author upon reasonable request.
