# Supplementary material for: Loss of KDM4B impairs osteogenic differentiation of OMSCs and promotes oral bone aging
Source: Int J Oral Sci. 2022 May 7;14:24. doi: 10.1038/s41368-022-00175-3 (PMC9079076; doi:10.1038/s41368-022-00175-3)
Supplement: Supplementary file 1 — Supplemental Material [file 41368_2022_175_MOESM1_ESM.pdf]

Supplementary data

Loss of KDM4B impairs osteogenic differentiation of OMSCs and promotes Oral Bone Aging

Running title: KDM4B controls oral bone aging

Fig. S1 Osteogenic and adipogenic differentiation potentials of BMSCs and OMSCs *in vitro*.

a ALP staining of BMSCs and OMSCs undergone osteogenic induction. Scale bar, 8 mm. *n* = 3.

b Quantitative ALP activity assay of BMSCs and OMSCs upon osteogenic induction. *n* = 3.

c ARS staining of BMSCs and OMSCs undergone osteogenic induction. Scale bar, 8 mm. *n* = 3.

d Quantification of calcium deposit in ECM of ARS staining of **c**. *n* = 3.

e Expression of osteogenic marker genes *Col1a1, Ibsp, Runx2, Sp7* and *Bglap* in BMSCs and OMSCs upon osteogenic induction. *$P < 0.05$; **$P < 0.01$. *n* = 3.

f-g Oil Red O staining of BMSCs and OMSCs upon adipogenic induction. Scale bar, 100 μm. *n* = 3.

h qRT-PCR analysis of the expression of adipogenic marker genes *CD36, Cebpa, Fabp4*, and *Pparg* in BMSCs and OMSCs upon adipogenic induction. **$P < 0.01$. *n* = 3.

Fig. S1

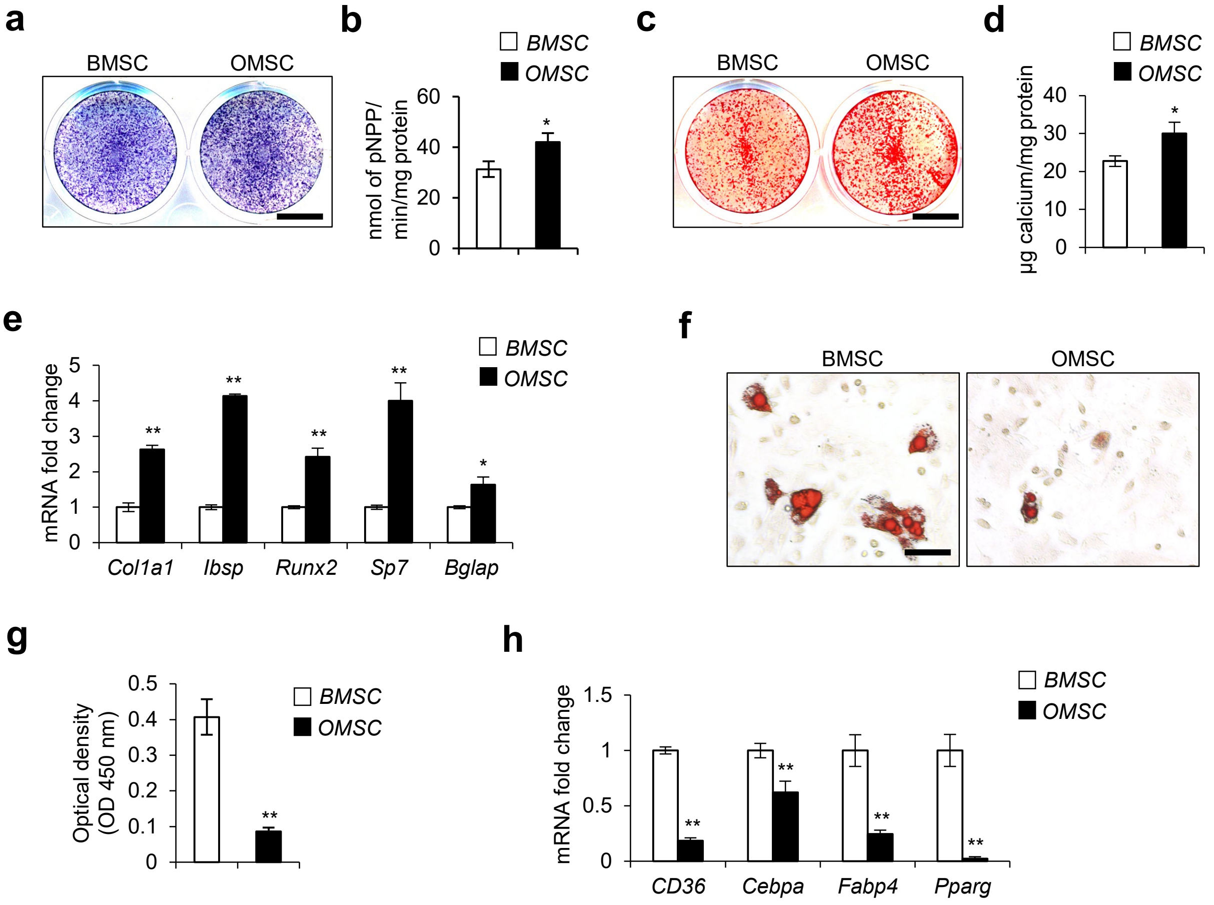

a
BMSC
OMSC
b
BMSC
OMSC
nmol of pNPP/min/mg protein
60
40
20
0
*
c
BMSC
OMSC
d
BMSC
OMSC
µg calcium/mg protein
40
30
20
10
0
*
e
BMSC
OMSC
mRNA fold change
5
4
3
2
1
0
**
**
**
**
*
Col1a1
Ibsp
Runx2
Sp7
Bglap
f
BMSC
OMSC
g
Optical density (OD 450 nm)
0.5
0.4
0.3
0.2
0.1
0
BMSC
OMSC
**
h
BMSC
OMSC
mRNA fold change
1.5
1
0.5
0
**
**
**
**
CD36
Cebpa
Fabp4
Pparg
